# Author Correction: Predicted reentrant melting of dense hydrogen at ultra-high pressures

**DOI:** 10.1038/s41598-023-38003-1

**Published:** 2023-07-18

**Authors:** Hua Y. Geng, Q. Wu

**Affiliations:** National Key Laboratory of Shock Wave and Detonation Physics, Institute of Fluid Physics, CAEP, P.O. Box 919-102, Mianyang, Sichuan 621900 P. R. China

Correction to: *Scientific Reports* 10.1038/srep36745, published online 11 November 2016

This Article contains errors in the Introduction, where the number of atoms in the primitive cell of both Cmcm and C222_1_ phases is incorrectly given as 8.

As a result,

“In particular, our results show that the previously proposed groundstate^16^ *Cmcm*-8 (a structure has the *Cmcm* space group symmetry with 8 atoms in the primitive cell) is thermodynamically unstable with respect to a newly discovered *C222*_*1*_-8 phase. The number of atoms in the primitive cell will not be given explicitly below for the sake of brevity. For example, *Cmcm-*8 will be abbreviated to *Cmcm* if there is no confusion.”

should read:

“In particular, our results show that the previously proposed groundstate^16^ *Cmcm*-6 (a structure has the *Cmcm* space group symmetry with 6 atoms in the primitive cell) is thermodynamically unstable with respect to a newly discovered *C222*_*1*_-6 phase. The number of atoms in the primitive cell will not be given explicitly below for the sake of brevity. For example, *Cmcm-*6 will be abbreviated to *Cmcm* if there is no confusion.”

In addition, Table 1, which lists the structural information of these two phases is absent in the original version of this Article.

Table 1 and accompanying legend appear below.

**Table 1:** The structure parameters for *Cmcm* (also denoted as oC12 in^1^) and *C222*_*1*_ phase. The length of lattice vectors is in a unit of Å.

Space group Pressure	Lattice parameters	Multiplicity	Coordinates
*C222*_*1*_ 3 TPa	(2.7952, 0.9479, 2.3372) (90.0°, 90.0°, 90.0°)	8H 4H	0.86418 0.12707 0.33189 0.39617 0.00000 0.00000
*Cmcm* 1.5 TPa	(1.0618, 3.0452, 2.6398) (90.0°, 90.0°, 90.0°)	4H 8H	0.00000 0.89778 0.25000 0.00000 0.36050 0.58603

## References

[1] Liu H, Wang H, Ma Y (2012). Quasi-molecular and atomic phases of dense solid hydrogen. J. Phys. Chem. C.

