# Supplementary material for: Predicted reentrant melting of dense hydrogen at ultra-high pressures
Source: Sci Rep. 2016 Nov 11;6:36745. doi: 10.1038/srep36745 (PMC5105149; doi:10.1038/srep36745)
Supplement: Supplementary Information [file srep36745-s1.pdf]

Predicted reentrant melting of dense hydrogen at ultra-high pressures

Hua Y. Geng* and Q. Wu

National Key Laboratory of Shock Wave and Detonation Physics, Institute of Fluid Physics, CAEP; P.O.Box 919-102, Mianyang, Sichuan, P. R. China, 621900

Supplementary Information

A. Compression effects on nearest-neighbor distance and pseudopotential

Usually compression reduces interatomic distance, and ultra-high compression might cause core overlap and leads to failure of the pseudopotential. However, the peculiarity of dense hydrogen is that the situation is just the opposite: the external pressure in fact compresses the inter-molecular space (or the van der Waals space) rather than the intra-molecular space, and the resultant dissociation of H_2 units leads to a larger nearest-neighbor (NN) distance, instead of the usually expected shortening of the NN distance. This unusual behavior of dense hydrogen has well been documented in Refs. [1,2]. In particular, the variation of the NN distance of monatomic hydrogen at ultra-high pressure regime has explicitly been given in the Fig. 14 of Ref. [2]. At higher pressures, for example the highest pressure of 7.3 TPa we have considered in this paper, the NN distances for the competing ground state of FCC and BCC phases are 0.78 and 0.76 Å, respectively. Both are slightly larger than the H_2 bond length of 0.74 Å at ambient conditions. This exceptional phenomenon guarantees that whenever a pseudopotential can be applied to low-pressure hydrogen, it also can safely be applied to dense hydrogen up to terapascal pressures. Figure 15 in Ref. [2] unambiguously demonstrated this by comparing the PAW results (from VASP) with the all-electron FLAPW results (from WIEN2k).

B. Size-effect and k-point convergence

It is well known that MD simulations with a small cell size would suffer from finite size effects, and might predict a higher melting temperature if the Z-method is employed. For dense hydrogen, there are a lot of MD simulations having been performed and reported in literature, which have established a good database for a

reliable estimate of the size effects in dense hydrogen. In the case of melting, Refs. [3-5] used 216, 432, and 1920 hydrogen atoms in the MD simulations, respectively. The perfect agreement of their obtained melting temperatures clearly indicates that the size effects are negligible when the simulation cell contains more than 200H atoms. This observation was further confirmed by detailed analysis given in Ref. [6]. Our simulation cells always contain 432-500H atoms, depending on the structure. They are large enough to eliminate possible size-effects. The perfect match of the melting temperatures calculated by the Z-method and the two-phase method justified this argument. Usually the former is much more prone to finite size effects and the latter is less sensitive to the cell size, as revealed by plentiful empirical experiences. On the other hand, our previous simulations showed that the convergence of the k-points is much more important than the size-effects for the cell size we used[4]. Figure S1 demonstrates the influence of different size of the k-point mesh on the melting temperature of Z-method. A good convergence is achieved with a 3×3×3 mesh, with an error of about 10 K in the resultant melting temperature.

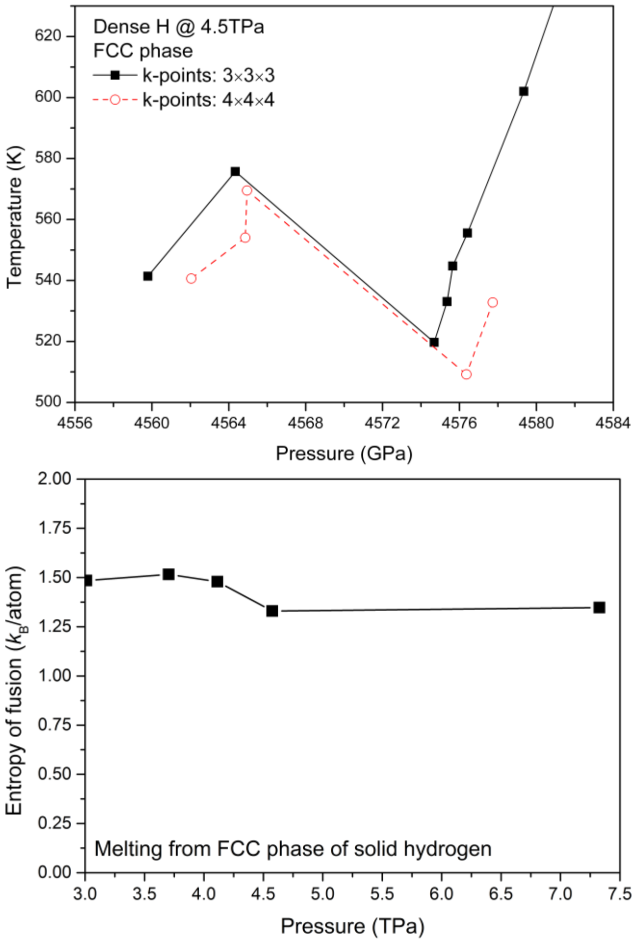

Figure S1 | (top) Typical Z-curves in the Z-method for melting modeling. The dense

hydrogen is at about 4.5 TPa heating from an FCC phase. The results of two different k-point meshes—3×3×3 and 4×4×4—are displayed. **(bottom) Entropy of fusion in dense hydrogen melting from FCC phase.**

C. Melting in Z-method

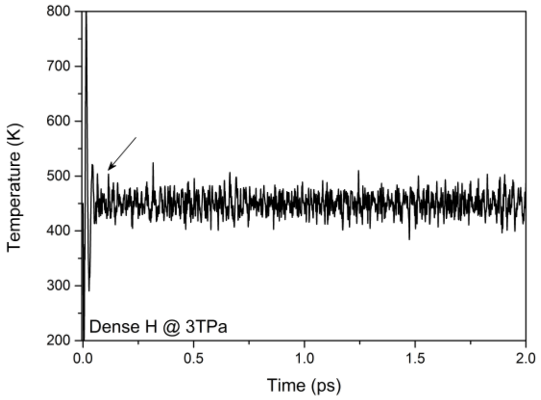

Figure S2 | Typical equilibrating time-scale in AIMD simulations of dense hydrogen. The structure is starting from the *Cmcm* phase at about 3 TPa, and equilibrates to the *C222*$_1$ phase at about 450 K with the Z-method. The arrow indicates the time when the system is fully equilibrated.

In AIMD simulations, to trigger a melting process is a rare event. It is thus crucial in Z-method that the system is fully equilibrated and is ergodicity before a melting point can be assessed. The equilibration in dense hydrogen is actually very fast. As shown in Fig. S2, the system equilibrates within 0.1 ps. This is due to the high vibrational frequency of hydrogen and the strong anharmonicity as revealed in Ref. [2]. The melting is initiated with a longer time, which requires about 0.25 ps (Fig. S3), but is much shorter than the typical time length of AIMD simulations we performed. The melting process leads to a redistribution of the energy between the potential and kinetic components, as revealed by the jumps shown in the Fig. S3.

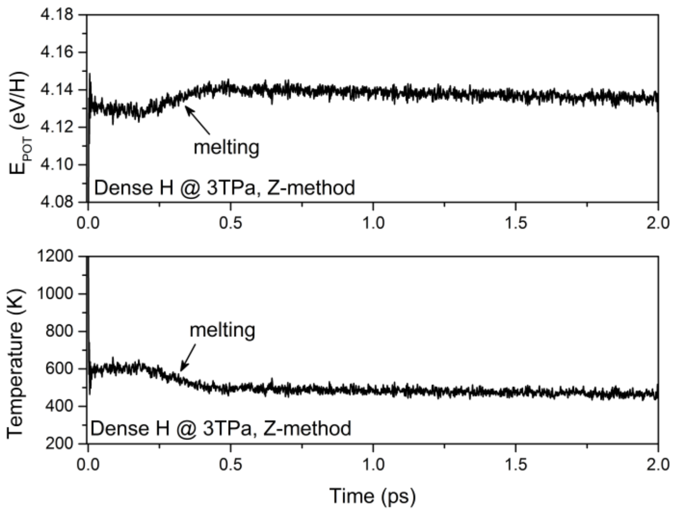

Figure S3 | Evolution of the potential energy and temperature when the system melts in Z-method. The structure is starting from the *Cmcm* phase at about 3 TPa, and equilibrates to the $C222_1$ phase, then melts into the liquid phase. The arrow indicates the time when the melt occurs.

The variation of the mean squared displacement (MSD) and radius distribution function (RDF) of dense hydrogen at 3 TPa along the Z-curve before and after the kink are shown in Fig. S4. A typical liquid behavior is observed at 470 K, which is immediately after the kink where the lattice collapses. Previous investigation revealed that dense hydrogen might transform into a mobile anisotropic state that is not a real "liquid"[4]. Here we analyzed the particle distribution carefully, and failed to find any anisotropy. This confirms that the transition is indeed a melting.

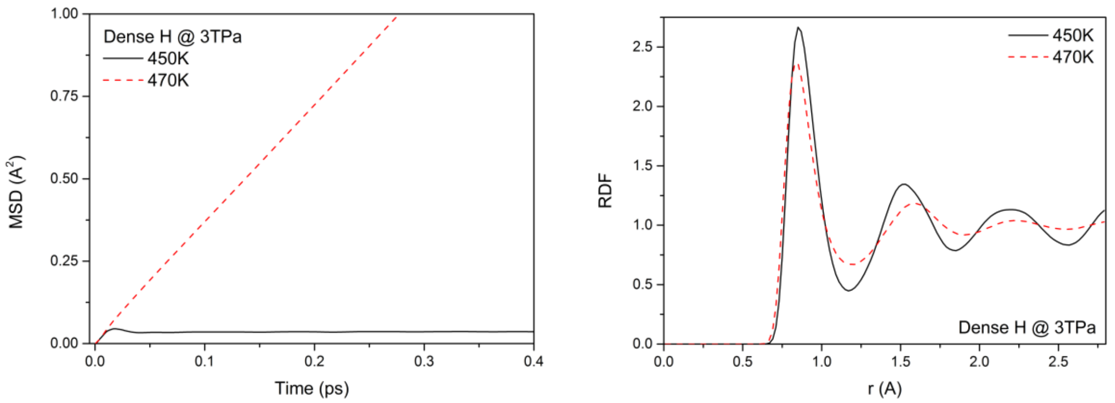

Figure S4 | Evolution of MSD (left) and RDF (right) with temperature in Z-method when crossing the kinked Z-curve. The structures are equilibrated before and after the occurring of the kink, respectively. The sharp variation of RDF and MSD from 450 to 470 K unambiguously indicates that it already melted at about 470 K.

D. *NPT* ensemble and two-phase method

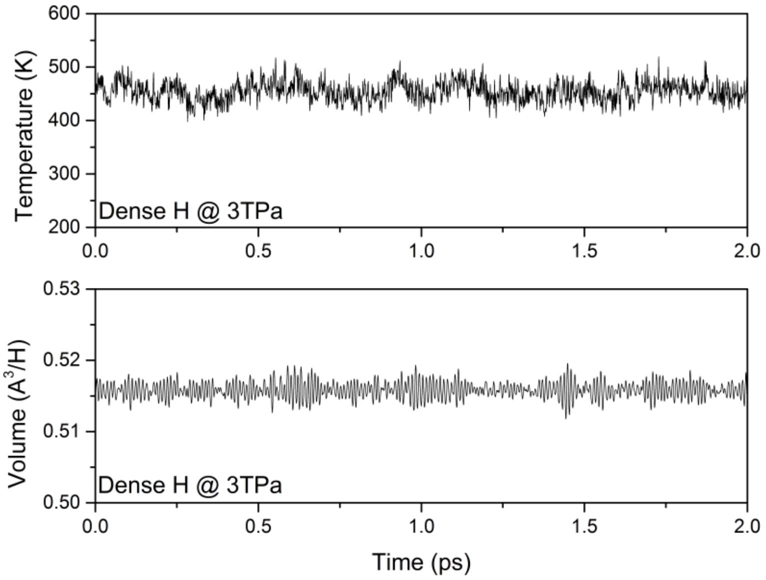

Figure S5 | Fluctuations of the volume and temperature in an AIMD simulation for modelling an *NPT* ensemble. The structure is equilibrated in the solid $C222_1$ phase at about 3 TPa. Efficient coupling with the thermostat and barostat is obvious.

The two-phase method in *NPT* ensemble is employed mainly for the purpose to check whether the Z-method overestimates the melting temperature or not. To achieve an *NPT* ensemble, the Parrinello-Rahman dynamics with Langevin thermostat as implemented in VASP is used. The fictitious mass and friction coefficient for lattice degrees of freedom are set to 20 atomic mass units and 55 ps^{-1}, respectively, and the friction coefficient for atomic degrees of freedom used in Langevin dynamics is set as 50 ps^{-1}. This setting leads to an efficient coupling with the thermostat and barostat, and equilibrates the system quickly, as indicated in Fig. S5. This good quality of AIMD simulations ensures a reliable two-phase modeling of the melting with *NPT* ensemble. A typical configuration of two-phase equilibration is illustrated by the snapshot shown in Fig. S6. It is necessary to point out that for the cell size we employed here, it is very difficult to maintain the two-phase equilibrating interface for a long time. Usually the system will evolve towards the solid or liquid phase quickly. Nonetheless, the uncertainty in the melting temperature introduced by this feature is less than 20 K, which is good enough for our purpose.

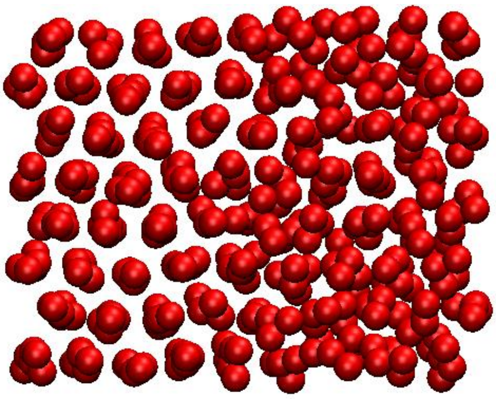

Figure S6 | A snapshot of two-phase equilibrating. The solid structure part is in the $C222_1$ phase, and the system is equilibrated at about 3 TPa and 460 K.

Supplementary References

[1] Labet, V., Hoffmann, R. & Ashcroft, N. W. A fresh look at dense hydrogen under pressure .III. Two competing effects and the resulting intra-molecular H-H separation in solid hydrogen under pressure. *J. Chem. Phys.* **136**, 074503 (2012); A fresh look at dense hydrogen under pressure. IV. Two structural models on the road from paired to monatomic hydrogen, via a possible non-crystalline phase. *J. Chem. Phys.* **136**, 074504 (2012).

[2] Geng, H. Y., Song, H. X., Li, J. F. & Wu, Q. High-pressure behavior of dense hydrogen up to 3.5 TPa from density functional theory calculations. *J. Appl. Phys.* **111**, 063510 (2012).

[3] Belonoshko, A. B., Ramzan, M., Mao, H. K. & Ahuja, R. Atomic diffusion in solid molecular hydrogen. *Sci. Rep.* **3**, 2340 (2013).

[4] Geng, Hua Y., Hoffmann, R. & Wu, Q. Lattice stability and high-pressure melting mechanism of dense hydrogen up to 1.5 TPa. *Phys. Rev. B* **92**, 104103 (2015).

[5] Liu, H., Zhu, L., Cui, W. & Ma, Y. Room-temperature structures of solid

hydrogen at high pressures. *J. Chem. Phys.* **137**, 074501 (2012).

[6] Chen, J. *et al.* Quantum simulation of low-temperature metallic liquid hydrogen. *Nat. Commun.* **4**, 2064 (2013).
